# Contemporary Issues in Head and Neck Pathology and Radiology

**DOI:** 10.1155/2010/621043

**Published:** 2011-03-02

**Authors:** Paul C. Edwards, Preetha P. Kanjirath, Tarnjit Saini, Neil S. Norton

**Affiliations:** ^1^Department of Periodontics and Oral Medicine, School of Dentistry, University of Michigan, 1011 N. University Avenue, Ann Arbor, MI 48109, USA; ^2^Office of Admissions, Midwestern University, 555 31st Street, Downers Grove, IL 60515, USA; ^3^US Army Dental Activity (DENTAC), Brooke Army Medical Center, Fort Sam Houston, San Antonio, TX 78234, USA; ^4^Department of Oral Biology, School of Dentistry, Creighton University, Omaha, NE 68178, USA

The comprehensive evaluation, assessment, and management of patients with non-tooth-related conditions of the head and neck area are essential aspects of the practice of dental medicine. The manuscripts selected for publication in this special issue serve to illustrate the importance of close cooperation between oral and maxillofacial pathology, radiology, oral medicine, and head and neck anatomy in both the initial diagnostic and subsequent treatment phases when evaluating and treating patients with non-tooth-related conditions of the oral and maxillofacial complex.

I would like to genuinely thank my Guest Editors, Dr. Neil S. Norton from Creighton University in Omaha, Neb, USA Dr. Preetha P. Kanjirath, from the University of Michigan at Ann Arbor, Mich, USA and Dr. Tarnjit Saini, Brooke Army Medical Center Fort Sam Houston, in San Antonio, Tex, USA for their assistance. Without their involvement and thoughtful discussions, this special issue would not have been possible. I also extend my thanks to the authors who have contributed to this special issue, as well as to the many reviewers who graciously volunteered with the peer-review process. 

In the lead article in this special edition, “*Bone Diseases of the Jaws*”, by P. J. Slootweg, provides an overview of the more common and/or important lesions occurring in the oral and maxillofacial complex, while emphasizing the considerable overlap in clinical, histological, and radiological features among these entities.

Y. Morimoto and colleagues review the usefulness of ultrasound imaging for the detection of noninvasive and soft tissue-related diseases and introduce three new potential applications of ultrasonography: guided fine-needle aspiration, measurement of tongue cancer thickness, and diagnosis of metastasis to cervical lymph nodes.

Subsequent manuscripts explore the relationship between craniofacial pathology and anatomy. L. Sonnesen summarizes recent studies on the link between morphological deviations of the cervical vertebral column and craniofacial morphology, while Guest Coeditor Neil S. Norton and colleagues employ volumetric tomography to review the anatomy of the greater palatine canal and also to rule out a statistically significant association between the prevalence of maxillary sinus disease and the presence of concha bullosa and/or nasal septal deviation.

R. A. Mesquita and colleagues critically review the available literature on the nonsurgical treatment of oral leukoplakia, while E. de S. Tolentino and colleagues present a well-documented case of an ameloblastic fibroma that illustrates the need to integrate radiology, oral and maxillofacial pathology, and head and neck anatomy in both the initial diagnosis and subsequent treatment of lesions of the maxillofacial complex.

On behalf of my Guest Coeditors and myself, I hope that you will find the manuscripts that comprise this special issue both interesting and informative.

*Paul C. Edwards* *Paul C. Edwards* *Preetha P. Kanjirath* *Preetha P. Kanjirath* *Tarnjit Saini* *Tarnjit Saini* *Neil S. Norton* *Neil S. Norton*

